
JOURNAL INFORMATION
==============================
NLM Title Abbreviation: Innov Surg Sci
Journal ID: Innov Surg Sci
NLM Journal ID: 101708165
Journal ID: iss

Innovative Surgical Sciences

EISSN: 2364-7485
Publisher: De Gruyter

ARTICLE INFORMATION
==============================
PMCID: PMC11917186
PMID: 40110108
DOI: 10.1515/iss-2025-2008
Article ID: iss-2025-2008
Article version: 1
Subjects: Article

Abstracts DGPRÄC

Electronic publication date: 2025 Mar 19
Publication date: 2025 Mar
Volume: 10
Issue: Suppl1
First page: 376
Last page: 381
Copyright: © 2025 by Walter de Gruyter Berlin/Boston
Copyright year: 2025
Copyright holder: De Gruyter
License: This work is licensed under the Creative Commons Attribution 4.0 International License.
License URL: https://creativecommons.org/licenses/by/4.0/

Page count: 6

==============================

Abstracts – DCK 2025 – 142nd Congress of the German Society of Surgery – Munich, March 26–28 • DOI 10.1515/iss-2025-2008
Innov Surg Sci 2025; 10 (Special Suppl 1): s376–s381

**Gender Career Gap in Pediatric Surgery - How to Break the Glass Ceiling and Fix the Pipeline**
ID: 602

**Kategorie: DGPRÄC - „Better Safe Than Sorry“ in der ästhetischen Chirurgie**

Hannah Luz[1], Julia Haehl[2], Sabine Zundel[1]

[1]*Kinderchirurgie, Kinderspital Zentralschweiz, Luzern, Schweiz*, [2]*Kinderchirurgie, Dr. von Haunersches Kinderspital, München, Deutschland*

**Background:**
Storms on social media like the #ILookLikeASurgeon movement that led to more than 128 million impressions across the globe as well as the #NYerORCoverChallenge have drawn attention to the lack of visibility of women in a profession traditionally dominated by men. In literature, this phenomenon is defined as the gender career gap, which is evident not only in pediatric surgery in general, but also in academic pediatric surgery. Women continue to be underrepresented in leadership positions in pediatric surgery.
These phenomenons are summarized by two main metaphors: the “glass ceiling effect“ and the “leaky pipeline phenomenon”. In view of an impending shortage of surgeons in the US and in Europe, “fixing the leaky pipeline” in pediatric surgery is not only necessary for individual satisfaction, but is primarily about ensuring that qualified staff are retained. We therefore asked ourselves where exactly the leaks in the pipeline are causing us to lose male and female pediatric surgeons and, above all, how they can be fixed to ensure the highest possible quality of care in our profession in the future.

**Materials and methods:**
We created a web-based online questionnaire through ScoSci survey, which comprised a maximum of 21 questions, with some questions being offered only depending on previous answers. The survey was distributed worldwide via email through the pediatric surgery associations as well as advertised on LinkedIn. This distribution led to 185 respondents participating in the survey (n = 185). Data analysis was performed through Microsoft Excel and Stata for the multiple-choice and short-text responses (that could be coded to integer data) and NVivo for open-ended questions.

**Results:**

1) Study Population
35% of the respondents were male and 75% female pediatric surgeons. The median age for male respondents was 43 years, while for female respondents, it was 36 years. The mean age of male participants was 46 years, compared to 37 years for female participants. The respondents hailed from diverse geographic regions: North America (6%), Africa (11%), Europe (68%), Latin America (3%) and Asia (11%).
2) Gender-Based Career Differences
Our study found that 74% of female respondents believed that gender impacts career opportunities, compared to only 32% of male respondents. Additionally, 77% of female surgeons felt disadvantaged by their gender, while 57% of male respondents perceived no difference, and 29% believed they benefited. These perceptions underscore a significant gender divide in the experience of career advancement, where women are more acutely aware of the barriers they face.
3) Sexual Discrimination and Harrasment
Although inequality in career chances and sexual discrimination and harassment are not the same and should not be mixed up, sexual discrimination and harassment are still a symptom for gender inequality and can be a reason for symptoms of burnout and suicidal thoughts. Being provided specific examples, a striking 93% of female respondents reported experiencing at least one instance of sexual discrimination, and 42% reported experiencing sexual harassment. In contrast, 50% of male respondents reported no experience of sexual discrimination, and 26% reported an experience of sexual harassment.

Open Access. © 2025 The Author(s), published by De Gruyter. This work is licensed under the Creative Commons Attribution 4.0 International License.

4) Parenthood and Career Progression
   Additionally, men were less likely to reduce work for childcare compared to female surgeons (71% vs. 29%). Among those who did take time off, women took significantly longer periods than men, reflecting the disproportionate caregiving responsibilities often assumed by women (mean 37.8 months vs. 9.9 months). Furthermore, a higher percentage of female surgeons reported that having children had altered their career goals, a finding that resonates with existing research showing that women in surgery frequently experience conflicts between their career ambitions and family responsibilities.
5) Possible Solutions
   Participants were explicitly asked about possible solutions on personal, institutional, and political levels. Most responses included improving conditions for parental leave both for male and female surgeons and possibilities for part-time work as well as the implementation of mentorship programs aimed at promoting women in pediatric surgery.

**Conclusion:**
Our findings illustrate the multifaceted nature of gender inequality in pediatric surgery, encompassing disparities in career advancement, academic achievement, perceptions of gender bias, experiences of discrimination and harassment, and the impact of parenthood. Addressing these issues requires a concerted effort to implement policies that support gender equity. Continued research and policy action are essential to closing the gender gap in pediatric surgery and ensuring that all surgeons can achieve their full potential, regardless of their gender.

### Necrotising fasciitis - therapy and strategies for plastic reconstruction
ID: 229

**Kategorie: DGPRÄC - Komplexe Rekonstruktion bei Verbrennung oder schweren Weichgewebsinfektionen**

Philipp Unbehaun[1], Vanessa Brebant[1], Lukas Prantl[1], Konstantin Frank[1], Alexandra Anker[1]

[1]*Caritas-Krankenhaus St. Josef, Abteilung für Plastische- und Ästhetische, Hand- und Wiederherstellungschirurgie, Regensburg, Deutschland*

**Background:**
Necrotizing fasciitis is a rare but life-threatening condition that requires specialized clinical infrastructure and experienced surgeons. Aggressive medical and surgical treatment, as well as hyperbaric oxygen therapy (HBO), is necessary. In this single-center study, we present our clinical experience as a center for necrotizing fasciitis over the past 10 years.

**Materials and methods:**
We retrospectively analyzed the data of 45 patients treated between 2014 and 2024. The study included patients who received intensive care treatment and had histologically confirmed necrotizing fasciitis. The surgical procedures, antibiotic treatments, pathogens, and laboratory parameters were evaluated.

**Results:**
The results showed a hospital mortality rate of 32%. The mean hospital stay was 44 days, with an average of 7 surgical procedures performed per patient. In 48% of the cases, the infections were classified as Type I, with *Escherichia coli* and *Staphylococci* being the most commonly identified pathogens. The average LRINEC score at the time of referral was 8, with a significant difference between the initial scores of deceased and surviving patients. For plastic reconstruction, most patients underwent skin grafting (n=17), while in 10 cases (20%), free flap surgeries were performed. Three patients underwent lower limb amputation.

**Conclusion:**
Eventhough successful treatment requires early aggressive debridement, following reconstructive options should already be considered. Hyperbaric oxygen therapy (HBO), and optimization of intensive care management are the further cornerstones. For antibiotic treatment, we routinely use a combination of piperacillin/tazobactam, clindamycin, and penicillin G, which has proven effective.

### Validation of the “Number Y” as a tool for Implant height selection and profile prediction in Breast Augmentation with Anatomical Prothesis in Gender affirming breast surgery

ID: 552

**Kategorie: DGPRÄC - Patientensicherheit in der Brustrekonstruktion**

Laetitia Chiarella[1 2 3], Isabel Kiehlmann[4], Tobias Hirsch[1 2 3], Charalampos Varnava[1 2 3], Marie-Luise Aitzetmüller-Klietz[1 2 3], Matthias Aitzetmüller[5], Ulrich M. Rieger[4], Sascha Wellenbrock[1 2 3]

[1]*Department of Plastic Surgery, Center for Transgender Health, University Hospital Muenster, Münster, Deutschland*, [2]*Department of Plastic and Reconstructive Surgery, Institute for Musculoskeletal Medicine, Münster, Deutschland*, [3]*Department of Plastic, Reconstructive and Aesthetic Surgery, Hand Surgery, Fachklinik Hornheide, Münster, Deutschland*, [4]*Department of Plastic and Aesthetic, Reconstructive and Hand Surgery, AGAPLESION Markus Hospital, Frankfurt am Main, Deutschland*, [5]*Department of Plastic, Aesthetic, Reconstructive and Hand Surgery, Klinikum Passau, Medical Campus of Lower Bavaria, Passau, Deutschland*

**Background:**

Breast augmentation is the second most commonly performed aesthetic procedure globally, with nearly 1.89 million procedures annually. Among individuals suffering from gender dysphoria, a condition affecting approximately 25 million people worldwide, there has been a significant rise in the demand for gender-affirming surgery (GAS), with procedures increasing fivefold over the past two decades. Research shows a correlation between GAS and improved mental health, enhanced quality of life, and better social integration. Chest feminization surgery with implants is the most frequently performed GAS for transfeminine patients. Proper implant selection is crucial for achieving optimal outcomes, taking into consideration factors like upper breast pole volume, implant height, somatotype, and breast footprint. (1) Recent studies suggest that the "Y-Number" could be a valuable tool for determining implant base, height, and projection in both cisgender and transgender patients. (2) This study aims to validate the use of the Y-Number in the transgender population, hypothesizing its effectiveness in predicting implant dimensions based on chest wall morphology.

**Materials and methods:**

Breast implant shapes are defined by key parameters: implant base, projection, width, height, and lower curvature. The implant base, corresponding to breast width, can be round or elliptic, while projection refers to how far the implant extends outward from the chest wall, with options such as low, moderate, and high, affecting the overall contour. This retrospective study included 24 patients who underwent breast reconstruction following gender affirmation surgery at AGAPLESION Markus Hospital in Frankfurt, Germany, between 2016 and 2021. Patients who received non-anatomical breast implants were excluded from the study (n=4). For each patient, the Y Factor was calculated using the method from del Yerro et al., which divides the thoracic perimeter by the suprasternal notch-to-nipple distance. The exposure variable compared pre- and intra-operative implant heights and base shapes (vertical, round, or elliptical) in transgender patients. A high Y-number indicated a horizontally elliptical shape with low projection. Discordance was noted when the Y-number-recommended projection differed from the actual implant chosen. Patient characteristics were assessed and compared by exposure. Continuous variables were analysed using ANOVA if normally distributed, or with the Wilcoxon Rank-Sum tests if not. Distribution was evaluated using the Kolmogorov-Smirnov test, and categorical variables were compared using Pearson's chi-squared test. All statistical analyses were conducted using STATA16, with a p-value of less than 0.05 considered statistically significant.

**Results:**

The average age of the cohort was 39.62 years (± 15.69), with a mean BMI of 25.42 (± 4.55). The average sternal notch-to-nipple (SNN) distance measured 21.65 cm (± 1.60 cm), and the mean thoracic perimeter was 93.17 cm (± 7.51 cm), resulting in an average Y-Number of 4.31 (± 0.32). Most patients (62%) underwent primary breast reconstruction, with 90.5% receiving implants with a horizontal-elliptic base. The average breast base width was 134.29 mm (± 7.79), the breast height averaged 114.71 mm (± 8.84), the anterior projection was 47.14 mm (± 6.37), and the lower ventricular curve measured 62.81 mm (± 5.72).

The majority of patients (66.67%) were recommended low-projection implants, though they more commonly received moderate (71.43%) or high-projection (23.81%) implants. Transgender patients undergoing gender-affirming surgery were found to have a higher Y-Number (mean 4.31 ± 0.32) compared to cisgender women.

The cohort was divided into concordant and discordant groups based on recommended and actual implant projections. Although discordant patients were older with higher BMI, these differences were not significant. SNN distance was similar between groups, but the discordant group had a significantly larger thoracic perimeter by 7 cm (P=0.04) and a higher Y-Number (4.47 ± 0.27 vs. 4.01 ± 0.15; P<0.001). Patients with concordant projections were mostly recommended moderate projections (86%), while those with discordant projections were primarily recommended low projections (93%) but received moderate (64%) or high (36%) projections instead. The anterior projection was significantly larger in the discordant group (49.14 ± 6.58 cm) compared to the concordant group (43.14 ± 3.62 cm, P=0.04). Both cohorts predominantly received horizontal-elliptic implants (86% vs. 93%).

**Conclusion:**
The Y-Number, as previously described, is not applicable to transgender women undergoing gender-affirming breast surgery. Further research is needed to develop an updated version of the Y-Number tailored to the transgender population.

(1) Nauta AC, Baltrusch KM, Heston AL, et al. Differences in Chest Measurements between the Cis-female and Trans-female Chest Exposed to Estrogen and Its Implications for Breast Augmentation. *Plast Reconstr Surg Glob Open*. 2019;7(3):e2167. Published 2019 Mar 13. doi:10.1097/GOX.0000000000002167

(2) Del Yerro JLM, Vegas MR, Fernandez V, et al. Selecting the implant height in breast augmentation with anatomical prosthesis: the "number Y". *Plast Reconstr Surg*. 2013;131(6):1404-1412. doi:10.1097/PRS.0b013e31828bd65b

### Outcomes of selective motor nerve transfer surgery in foot drop
ID: 399

**Kategorie: DGPRÄC - Plexus- und periphere Nervenchirurgie - motorische Ersatzoperationen**

Judith Döringer[1], Dominique Merky[1], Samuel Christen[2], Esther Vögelin[1], Rémy Liechti[1]

[1]*Inselspital Bern, Bern, Schweiz*, [2]*Berit Klinik, Goldach, Schweiz*

**Background:**
This retrospective analysis evaluates the functional, morphological (ultrasound) and electrophysiological outcomes following selective motor tibial nerve branch transfer to the deep peroneal motor nerve branch of the anterior tibial muscle in patients with foot drop. The procedures were performed at the University Hopital of Bern, Switzerland.

**Materials and methods:**
All consecutive patients undergoing selective motor nerve transfer (sMNT) for foot drop with or without combined neurolysis of the peroneal nerve between January 2011 and March 2023 were assessed for eligibility. Patients younger than 16 years, those with less than 9 months of follow-up, those unwilling to participate and those with concomitant peripheral nerve conditions were excluded from the analysis.

**Results:**
sMNT surgery was performed on 21 patient, with an average interval of 7.2 months between trauma and surgery (range 0-15 months). Of these, 17 patients met the inclusion criteria, 13 of which underwent concomitant peroneal nerve neurolysis. The average age was 42.8 years (range 16-72 years), with a mean follow-up of 27.5 months (range 6-63 months) post-surgery. Reinnervation was observed in 14 patients (82.3%) at an average of 6.0 months after surgery, with the tibialis anterior (TA) muscle strength improving by an average of 3.6 points (SD 1.3; range 1-4.75) on the Medical Research Council (MRC) scale. 12 of the 17 patients (70.6%) were able to walk without an orthosis, 10 of whom demonstrated an MRC grade of 4 or higher. These patients had significantly shorter reinnervation times compared to weaker patients (7.6months, SD 8.0 vs 21.8 months, SD 14.7; $p=0.028$) . Electromyography (EMG) revealed notable improvements in the amplitude of muscle unit potentials in the TA muscle. Motor recovery of the TA muscle was closely associated with patient age, number of transferred fascicles and combined neurolysis of the peroneal nerve.

**Conclusion:**
sMNT surgery appears to be a viable option for restoring functional ankle dorsiflexion in patients with foot drop. Combined neurolysis of the peroneal nerve seems to enhance outcomes. Additionally, the number of transferred fascicles and MRC scores correlate, suggesting that sMNT plays a key role in motor reinnervation.
